# An open-source SQL database schema for integrated clinical and translational data management in clinical trials

**DOI:** 10.1177/17407745241304331

**Published:** 2024-12-25

**Authors:** Umar Niazi, Charlotte Stuart, Patricia Soares, Vincent Foure, Gareth Griffiths

**Affiliations:** 1 Cancer Research UK Southampton Clinical Trials Unit, MP131, Southampton General Hospital, University of Southampton, Southampton, UK; 2 iSolutions, University of Southampton, Southampton, UK

**Keywords:** Open-source SQL database schema for clinical trials, clinical trial data integration with translational data, personalised medicine in clinical research

## Abstract

Unlocking the power of personalised medicine in oncology hinges on the integration of clinical trial data with translational data (i.e. biospecimen-derived molecular information). This combined analysis allows researchers to tailor treatments to a patient’s unique biological makeup. However, current practices within UK Clinical Trials Units present challenges. While clinical data are held in standardised formats, translational data are complex, diverse, and requires specialised storage. This disparity in format creates significant hurdles for researchers aiming to curate, integrate and analyse these datasets effectively. This article proposes a novel solution: an open-source SQL database schema designed specifically for the needs of academic trial units. Inspired by Cancer Research UK’s commitment to open data sharing and exemplified by the Southampton Clinical Trials Unit’s CONFIRM trial (with over 150,000 clinical data points), this schema offers a cost-effective and practical ‘middle ground’ between raw data and expensive Secure Data Environments/Trusted Research Environments. By acting as a central hub for both clinical and translational data, the schema facilitates seamless data sharing and analysis. Researchers gain a holistic view of trials, enabling exploration of connections between clinical observations and the molecular underpinnings of treatment response. Detailed instructions for setting up the database are provided. The open-source nature and straightforward design ensure ease of implementation and affordability, while robust security measures safeguard sensitive data. We further showcase how researchers can leverage popular statistical software like R to directly query the database. This approach fosters collaboration within the academic discovery community, ultimately accelerating progress towards personalised cancer therapies.

Oncology research thrives on a collaborative approach. Clinical trials assess the effectiveness and safety of new therapies in humans, while translational research translates basic science discoveries into clinical applications. Clinical trials generate rich ‘clinical data’ including detailed patient demographics, disease characteristics, treatment details and outcomes. Later-phase trials may also collect quality-of-life and health economic data. Increasingly, trials incorporate ‘translational data’ from biological samples (or sometimes routine National Health Service data) – for example, molecular and genetic information. This data collection can occur at various points: Baseline: as part of eligibility criteria or patient stratification during randomisation; During the trial: as markers influencing treatment decisions (e.g. ctDNA or immune response as a secondary endpoint); Post-trial analysis: examining collected samples for pre-determined subgroup analyses or to explore disease mechanisms and treatment effects. This often informs future biomarker-driven trials. Southampton Clinical Trials Unit is a UK Clinical Research Collaboration registered Clinical Trials Unit and core funded by Cancer Research UK with the aim of ‘discovery is at the heart of everything we do’. Therefore, integrating these clinical and translational datasets for trial participants offers a powerful resource to the wider scientific community. By correlating clinical observations with underlying molecular alterations, researchers can investigate disease mechanisms at a deeper level. This approach can help identify factors influencing treatment response and ultimately tailor therapies to specific patient subgroups based on their unique molecular makeup. Personalised medicine holds immense promise for improving treatment efficacy, reducing side effects, and avoiding unnecessary treatments for non-responders.

In the United Kingdom, a trial unit’s clinical data are usually collected and managed in cloud-based Electronic Data Capture systems in a standardised format. However, data from translational research are usually complex and multi-dimensional, existing in different formats, coming from disparate sources and often needing specialised storage considerations.^1^ Furthermore, there is disparity in the timelines of data creation; the vast majority of translational data associated with trials are often generated a lot later in the timeline of the trial, in some cases after completion of clinical data collection, often using technology and knowledge that the discovery community did not have when the trial was designed and initiated 5–10 years earlier. Translational data also need to be interpreted in the context of its metadata, to account for confounders such as batch effects, for example. This metadata is often not adequately captured. The challenges faced by researchers thus include the management and analysis of translational datasets and associated metadata and the integration of translational data with clinical datasets.

A further consideration and challenge is in the onward data sharing for the purposes of further research. Cancer Research UK has a default position of open data sharing to the academic discovery community to accelerate progress in cancer research. Being part of the Cancer Research UK infrastructure, the Southampton Clinical Trials Unit is committed to this mission, fostering a more efficient and collaborative research landscape, ultimately leading to faster advancements in cancer prevention, diagnosis and treatment.

UK Clinical Trial Units often manage a portfolio of clinical trials, holding and managing large quantities of clinical and translational data. For example (see Figure 1), the Cancer Research UK-funded CONFIRM^2^ trial in mesothelioma (NCT03063450) consisted of 161 clinical data variables across 336 patients and several clinical visits including screening, baseline, treatment and post-treatment generating over 153,000 clinical data fields. Translational data encompasses a diverse range of formats and sizes. For instance, RNA-Sequencing (RNA-Seq) experiments generate massive raw genomic sequence files in FASTQ format (Level 0 data). These files require significant storage space due to their large size. After undergoing processing through a bioinformatics pipeline, they are transformed into a more manageable and informative format: a gene expression matrix (Level 1 data). This matrix summarises the activity of thousands of genes across multiple samples, providing valuable insights into biological processes. To illustrate, in the CONFIRM trial, a batch of 50 RNA-Seq data files in FASTQ format across 25 patients at baseline visit requires around 70 GB of storage space (Level 0 data). After processing through a bioinformatics analysis pipeline, these data are condensed into a gene expression profile matrix containing information for over 50,000 genes measured in each of the 25 patient samples (Level 1 data). Using our CONFIRM trial of 336 patients as an example, this would require an estimated storage space of just under 1 TB for the raw FASTQ files.

**Figure 1.**
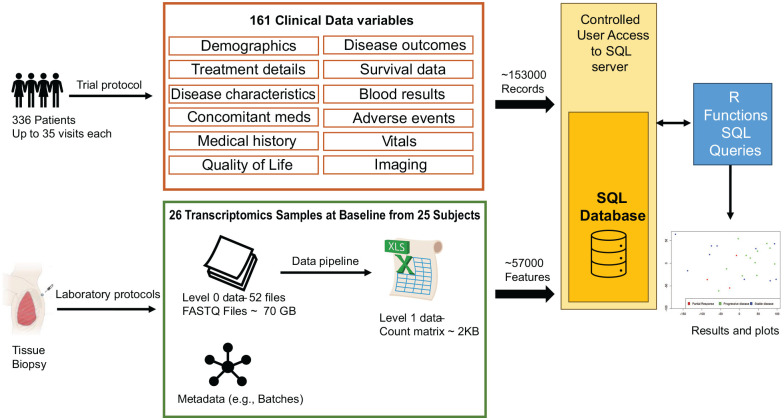
This infographic showcases how the CONFIRM clinical trial data and a specific set of translational data are integrated within a SQL database. Clinical Data: The study collected 161 different clinical data points from 336 patients across several visits, resulting in over 150,000 unique records stored in the database. Translational Data (Example): This example focuses on a batch of baseline transcriptomics data obtained from tissue biopsies. Samples: From a batch of 25 patients; Raw Data: 52 FASTQ files requiring approximately 70 GB of storage space (Level 0 data); Processed Data: Bioinformatics analysis transformed the raw data into a count matrix (Level 1 data) occupying roughly 2 KB and containing 57,000 features. Integration and Analysis: The strength of the database lies in its ability to connect all this information in one central access-controlled location. This enables researchers to perform comprehensive analyses that combine both clinical and translational data. The example plot visualises this integration. It displays the first two principal components of the processed transcriptomics data (Level 1), coloured by the disease outcome status retrieved from the clinical data. This exemplifies the real-time integration facilitated by the database.

Combining data across trials, for example, within a common disease area or tumour type, can increase the power of further translational analysis. However, several challenges impede this goal. Data Silos: historically, data from individual trials often become siloed due to their origin from diverse sources and application of trial-specific analysis pipelines. This lack of standardisation hinders dataset integration across multiple trials. Complexity: translational data, unlike standardised clinical data, exists in complex and multi-dimensional formats. Effective integration requires solutions that address this complexity, such as specialised data warehousing and management tools. Resource constraints: managing and analysing large datasets, especially when integrating data from multiple trials, can strain computational resources. This requires careful planning and infrastructure investment in high-performance computing capabilities. Furthermore, skilled personnel are crucial. Medical bioinformatics staff with expertise in statistics and data science are essential for navigating complex data formats, developing robust analysis pipelines and ensuring data quality. The lack of such expertise can significantly hinder the efficiency and effectiveness of data integration efforts. Permissions: data sharing agreements and access control mechanisms need to be established to ensure responsible data use while complying with ethical considerations.

The rise of Secure Data Environments/Trusted Research Environments such as the National Health Service Research Secure Data Environment Network or The Scottish National Safe Haven offers a solution to the problems described above; however, they do so at a cost that academic Clinical Trial Units often cannot always entertain. Furthermore, the data held in a Secure Data Environment need to be highly curated before it is uploaded, often a monumental task for historical and/or non-standardised data. Although Clinical Trial Units will and do use such secure environments for a number of well-funded trials or sharing with industry partners, there is a need for academic units to have a pragmatic ‘halfway-point’ solution between raw data and a Secure Data Environment which is practical and affordable for sharing data with an academic discovery community.

This article describes an open-source SQL database schema (Figure S1 and Table S1), licenced under the General Public License version 3.0, which offers a central unified hub for both clinical and translational data. Our design is informed by our experience managing Cancer Research UK trials, such as the CONFIRM mesothelioma trial, conducted in collaboration with the National Institute for Health and Care Research Biomedical Research Centre Leicester and their translational research focus on mesothelioma. For academic units, this offers a cost-effective solution and facilitates compliance with the FAIR principles (Findability, Accessibility, Interoperability and Reusability).^3^ While prioritising data security and anonymisation, integrating these datasets within a single platform allows authorised researchers to synchronise data, ensure a consistent timeline for all data points, facilitating a more holistic view of the trial, and unify analysis, enable seamless exploration of connections between clinical observations and molecular underpinnings of treatment response. While the core database schema is open-source, trial units can offer additional support services, such as bioinformatics and statistical expertise, to assist discovery researchers in effectively utilising and analysing the data.

The database schema is generalised allowing integration from multiple studies in a standardised format. Its open-source nature eliminates licencing fees, and the relatively straightforward design allows for easier implementation within existing IT infrastructure of a trials unit. This database also seamlessly integrates with statistical software like R.^4^ By leveraging SQL libraries within R, researchers can query and import the clinical and translational data directly for in-depth analysis. Furthermore, the database allows for results from these analyses to be uploaded back into the system, keeping the data continuously updated and accessible for future studies. For example, while doing a statistical analysis, new derived features may be constructed from a set of clinical features, which can be added as new entries to the Clinical Data table. This two-way communication between the SQL database and R software empowers researchers to conduct efficient analyses while maintaining a centralised and well-organised data repository. The SQL database enforces robust security measures to protect sensitive clinical and translational data which include user authentication and authorisation, user access controls, and data encryption. It should be noted that authorised users still have the ability to export data and results outside of the SQL server, and as a result, it is recommended to have a data sharing agreement (or appropriate equivalent) in place with all external users.

In conclusion, this article presents an open-source SQL database schema specifically designed to overcome the challenges of integrating and analysing clinical and translational data in cancer research. Informed by our experience managing trials like CONFIRM and aligning with the National Institute for Health and Care Research Biomedical Research Centre’s translational focus on mesothelioma, this schema offers a cost-effective and FAIR-compliant solution for academic trials units.

The open-source nature of the schema empowers researchers to readily adopt and utilise this framework within their own research groups. The comprehensive supplementary section details the design and implementation process and also provides link to an additional online guide containing a walkthrough for setting up an SQL Server instance, along with SQL scripts to create the database and example R code for common data input and extraction tasks. While the core schema is open source, bioinformatics support from clinical trial units can be instrumental in effectively implementing and utilising this framework, particularly for researchers with limited experience in database management and complex data analysis. By having discovery at the heart of everything we do and fostering data sharing and integrated analysis, this approach has the potential to significantly accelerate progress in cancer research, ultimately leading to the development of more effective and personalised therapies for patients.

## Supplemental Material

sj-pdf-1-ctj-10.1177_17407745241304331 – Supplemental material for An open-source SQL database schema for integrated clinical and translational data management in clinical trials Supplemental material, sj-pdf-1-ctj-10.1177_17407745241304331 for An open-source SQL database schema for integrated clinical and translational data management in clinical trials by Umar Niazi, Charlotte Stuart, Patricia Soares, Vincent Foure and Gareth Griffiths in Clinical Trials
