# Supplementary material for: An open-source SQL database schema for integrated clinical and translational data management in clinical trials
Source: Clin Trials. 2024 Dec 25;22(3):374–7. doi: 10.1177/17407745241304331 (PMC12092935; doi:10.1177/17407745241304331)
Supplement: sj-pdf-1-ctj-10.1177_17407745241304331 – Supplemental material for An open-source SQL database schema for integrated clinical and translational data management in clinical trials [file sj-pdf-1-ctj-10.1177_17407745241304331.pdf]

Supplementary Section: An Open-Source SQL Database Schema for Integrated Clinical and Translational Data Management in Clinical Trials- perspective of a Cancer Research UK clinical trials unit

This section delves deeper into the design and implementation details of the open-source SQL database schema presented in the main manuscript. It outlines the framework for managing and analysing integrated clinical and translational data in cancer research. For researchers seeking a hands-on guide to set up the database, a comprehensive walkthrough is available in the accompanying online repository: https://github.com/uhkniazi/SCTU_SQLDB_Supplementary.

Schema Design

The core schema comprises several relational tables designed to be general and capture specific data elements relevant to cancer studies (Figure S1 & Table S1). The schema centres around the **Study table**, which serves as the foundation, capturing overarching information about the trial. Linked to the Study table is the **Subjects** table. This table stores data pertaining to each unit of analysis, which is essentially a combination of a unique subject identifier and a specific visit number. The **Clinical Data** table establishes a bridge, associating each unit of analysis with its corresponding clinical data points.

For translational research aspects, the schema utilises the **Dataset** table. This table represents each distinct set of data generated from translational experiments. The **Omics Sample** table delves deeper, meticulously tracking individual samples within each dataset. These samples are then linked back to the **Subjects** table, ensuring traceability. Finally, the **Metadata** table plays a crucial role by capturing detailed laboratory metadata associated with each omics sample entry. This metadata encompasses vital information such as processing batch and sample quality metrics. Furthermore, the Omics Sample table establishes a connection to the **Level 0 Data** table. This table maps the raw, unprocessed data in its original format, like the FASTQ files containing the raw sequence reads directly from a sequencing machine after a genomics experiment. A companion **Level 1 Data** table exists within the schema. This table houses all the samples in a dataset after undergoing initial processing steps within a bioinformatics pipeline. In genomics, this could be a count matrix summarising the number of reads for each gene or genetic variant, derived from the raw FASTQ files. Each dataset in the **Dataset** table links to the corresponding processed data contained within the **Level 1 Data** table.

This structure fosters a comprehensive and interconnected data ecosystem, facilitating seamless integration of clinical and translational data within the broader context of the research study.

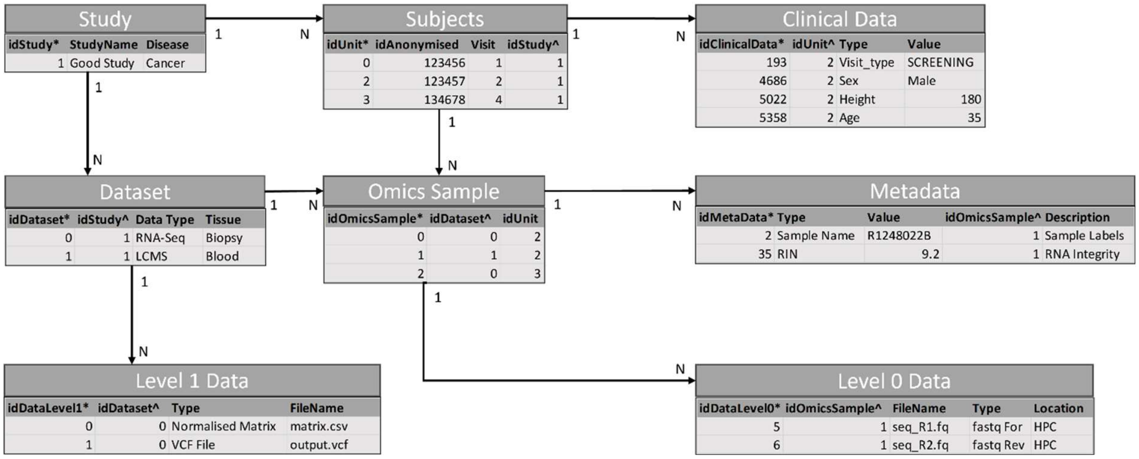

Figure S1: This diagram serves as a blueprint for the database schema, designed to integrate clinical and translational data across diverse research studies. Key entities and their relationships are described in detail within Table S1. Tables: Represent core data structures within the database. Attributes: Denote specific data fields within each table. Primary keys are marked with an asterisk (*), while foreign keys are indicated by a caret (^). Relationships: Illustrated by lines connecting tables. Arrows depict the direction of the relationship (one-to-many). Sample Data: Provided for illustrative purposes, allowing readers to visualize the database in action. Field Names: Shortened for enhanced readability of the diagram.

These tables are interconnected using **foreign keys** to ensure data integrity and facilitate efficient data retrieval and analysis. Foreign keys are essentially reference columns that link records in one table to another. This web of foreign key relationships ensures data consistency and allows researchers to efficiently query and analyse data across different aspects of a clinical trial and its associated translational research.

Table S1. Core Tables and Relationships in the Schema (see with Figure S1)

Table Name	Description	Key Attributes & Relationships
Study	Stores essential information about the clinical trial, including study name, disease focus, treatment arms, funding source, trial status, abstract describing the trial, date trial started, and location of trial documents.	idStudy (primary key), StudyName, Disease, Treatment, Funder, Status, Abstract, Date, and DocumentsLocation.
Subjects	Captures information about each visit from a study participant, including anonymised ID, visit number, and linkage to the specific study.	idUnit (primary key), idAnonymised (anonymised ID), Visit (visit number), and idStudy (foreign key referencing idStudy in the Study table – One to Many).

Clinical Data	Houses patient-specific clinical data collected throughout the trial, encompassing vital signs, laboratory results, imaging reports, response assessments, and adverse events.	idClinicalData (primary key), Type (Clinical data type), Value (Actual measurement for the Type), idUnit (foreign key referencing idUnit in the Subject table – One to Many), and Description.
Dataset	Holds information about omics datasets generated from the study, including data type, tissue source, date dataset was generated, analysis instrument, and processing steps.	idDataset (primary key), idStudy (foreign key referencing idStudy in the Study table – One to Many), OmicsDataType, Tissue, Git (link to Online Git repository), Date, Instrument, and ProcessingSteps.
Omics Sample	Links omics samples to the metadata, specific datasets and study participants.	idOmicsSample (primary key), idDataset (foreign key referencing idDataset in the Dataset table – One to Many), and idUnit (foreign key referencing idUnit in the Subject table – One to Many).
Level 0 Data	Stores details about raw data files associated with omics samples, such as file name, type, and location.	idDataLevel0 (primary key), idOmicsSample (foreign key referencing idOmicsSample in the OmicsSample table – One to Many), FileName, Type, and Location.
Level 1 Data	Contains processed omics dataset in a self-contained format (typically MIAME compliant count matrix), ready for further analysis.	idDataLevel1 (primary key), idDataset (foreign key referencing idDataset in the Dataset table – One to Many), FileName, Type, Location, and ProcessingSteps.
Metadata	Captures metadata associated with omics samples, such as sequencing lane, batch information, or RNA quality metrics.	idOmicsSampleMetaData (primary key), Type, Value, idOmicsSample (foreign key referencing idOmicsSample in the OmicsSample table – One to Many), and Description

Implementation Considerations

Data Types: The schema utilises appropriate data types for each column such as INT for numeric identifiers, VARCHAR for text strings and DATE for dates. Large files like trial protocol documents and non-text format data files (e.g. Omics count matrices) are not stored in the database, but instead a link is provided to the location on a separate storage system. This ensures data integrity and efficient storage.

Constraints: Primary key constraints are applied to each table to guarantee unique identification of each record. Additionally, unique constraints are used on specific combinations of columns (e.g., "idAnonymised", "Visit" and "Study_idStudy" in the "Subjects" table) to prevent duplicate entries.

Normalisation: The schema is designed to be in first normal form, ensuring that each column contains atomic data (single values) and avoiding redundancy. This simplifies data manipulation and retrieval.

In conclusion, by carefully considering these implementation details, the database schema presented here offers a robust and flexible framework for managing and analysing integrated clinical and translational data in cancer research. The schema's well-defined structure, combined with the comprehensive supplementary materials and online repository, empowers researchers to readily adopt and utilise this framework within their own research groups. This approach has the potential to significantly accelerate progress in cancer research, ultimately leading to the development of more effective and personalised therapies for patients.
